# Deep Learning 1D-CNN-Based Ground Contact Detection in Sprint Acceleration Using Inertial Measurement Units

**DOI:** 10.3390/s26010342

**Published:** 2026-01-05

**Authors:** Felix Friedl, Thorben Menrad, Jürgen Edelmann-Nusser

**Affiliations:** 1 Sports and Technology, Institute of Sport Science, Otto von Guericke University Magdeburg, 39116 Magdeburg, Germany; 2 Department Team, Combat & Acrobatic Sports, Institute for Applied Training Science, 04109 Leipzig, Germany

**Keywords:** deep learning, convolutional neural network, time-series classification, sprint acceleration, spatiotemporal, biomechanics, ground contact detection, sports performance analysis, motion analysis, field-based monitoring, Inertial measurement units, IMU

## Abstract

**Highlights:**

**What are the main findings?**

- A 1D-CNN accurately detected 100% of ground contacts during sprint acceleration.
- Ground contact times and event estimates were as accurate or better than previous methods.

**What are the implications of the main findings?**

- Deep learning 1D-CNN provides superior accuracy and reliability for ground contact detection in sprint acceleration.
- Deep learning approaches should be implemented in real-time field systems to enhance practical performance analysis.

**Abstract:**

**Background:** Ground contact (GC) detection is essential for sprint performance analysis. Inertial measurement units (IMUs) enable field-based assessment, but their reliability during sprint acceleration remains limited when using heuristic and recently used machine learning algorithms. This study introduces a deep learning one-dimensional convolutional neural network (1D-CNN) to improve GC event and GC times detection in sprint acceleration. **Methods:** Twelve sprint-trained athletes performed 60 m sprints while bilateral shank-mounted IMUs (1125 Hz) and synchronized high-speed video (250 Hz) captured the first 15 m. Video-derived GC events served as reference labels for model training, validation, and testing, using resultant acceleration and angular velocity as model inputs. **Results:** The optimized model (18 inception blocks, window = 100, stride = 15) achieved mean Hausdorff distances ≤ 6 ms and 100% precision and recall for both validation and test datasets (Rand Index ≥ 0.977). Agreement with video references was excellent (bias < 1 ms, limits of agreement ± 15 ms, r > 0.90, *p* < 0.001). **Conclusions:** The 1D-CNN surpassed heuristic and prior machine learning approaches in the sprint acceleration phase, offering robust, near-perfect GC detection. These findings highlight the promise of deep learning-based time-series models for reliable, real-world biomechanical monitoring in sprint acceleration tasks.

## 1. Introduction

In cyclic sports such as general running and more intense short-distance sprinting, spatiotemporal parameters have long been a major focus of research, with ground contact (GC) characteristics being among the most relevant [1,2]. The athletic 100 m sprint comprises distinct phases, and in each of them, GC time plays a crucial role in determining performance [3]. Beyond their intrinsic biomechanical significance, GC events serve as temporal reference points that enable the identification and synchronization of additional biomechanical parameters characterizing athletic performance [1,2,4,5]. A GC is hereby defined as the interval between two events: the initial contact (IC) of the foot with the ground and its subsequent toe-off or terminal contact (TC) [5]. GC events are measured with a variety of systems, including insole pressure sensors, embedded force platforms, consumer-grade cameras, high-speed cameras, photoelectric bars, or inertial measurement systems (IMUs) [5]. IMUs are small, portable, and wireless wearables containing accelerometers and gyroscopes [2]. Assessing running gait in a laboratory setting often does not reflect real-world scenarios, and IMUs advantageously offer a good way to assess sprinting in field conditions [6,7,8]. However, IMUs are still used mainly indoors, on a treadmill at prescribed speeds, or over small distances only [6]. Recent studies found that the accuracy of GC time detection using IMU is affected by the speed of movement [5]. In sprinting, especially in the phase of acceleration, caution is warranted, as IMU GCs can show reduced validity and reliability [9]. A review of Mendicino et al. (2025) found 37 studies on IMU GC detection in high-impact sports; of these, 28 examined running, and only 3 focused on athletic sprinting tasks [5].

The 100 m track sprint can generally be divided into different phases: the start, an acceleration phase, a phase of maximum speed, and a deceleration phase [10]. Traditionally, the acceleration phase has been described as spanning from approximately zero up to 30–50 m [11]. More recent studies split the acceleration phase into an initial acceleration from 0 to 10 m and an acceleration transition phase from 10 to 30 m [10,12]. Recent research has examined acceleration on a step-specific level. Acceleration changes from the initial 1st–3rd step phase (initial acceleration), characterized by mostly step-frequency adaptations; to the 5th to 14th–16th step (acceleration transition), a phase focused on step-length increase; and then to the 16th step onwards (maximum speed), when the trunk reaches its most upright position [13,14,15,16,17]. The acceleration is generally the phase where kinematic stride parameters change most dynamically in a sprint, which influences GCs [17]. In the 1st step, the GC accounts for 77.4% of the stance, which decreases until the 8th step, where flight time and GC time are balanced [17].

To obtain GCs from IMUs, Mendicino et al. (2025) found 71 different detection algorithms in their literature review, from which 67 relied on heuristic or rule-based algorithms and 4 on machine or deep learning approaches [5]. Heuristic and rule-based algorithms claim that different maxima, minima, and thresholds in IMU data, sometimes within certain time windows, represent the IC and TC [18]. Caution must be taken with the accuracy of heuristic or rule-based algorithms as the minima, maxima, thresholds, and time windows may fluctuate due to athlete-specific anthropometry, movement techniques [19], the segment of IMU attachment, and varying and changing speed levels [5]. Therefore, the claim that heuristic models exactly represent IC and TC may not be true for all steps, which can be advantageous for machine or deep learning approaches, where the GC events can be trained directly with a validation measurement system.

Applying heuristic algorithms, Blauberger et al. (2021) found mean absolute GC time deviations of 5.46 ± 4.55 ms in elite sprinters up to 100 m runs [20]. The algorithm failed to detect 6.47% of the GC in the initial acceleration phase (first five steps) while performing better in other running phases (0.56%) except the last five steps (13.33%). Another study of maximum accelerated sprinting (0–10 m) found mean deviations of −71 ± 70.3 ms and limits of agreement (LOA) with a 93% GC detection rate [21]. Comparing IMUs against force plates in 50 m maximum accelerated sprinting, Van den Tillaar et al. observed significant differences with a mean bias of 3 ms (moderate correlation) and 4 ms (trivial correlation) using IMU sampling at 1000 Hz and 240 Hz, while no information on LOA or detection failure rate was given [22]. Purcell et al. (2006) reported −8 ± 17.64 ms for the first step, −2 ± 9.8 ms for the third, and 0 ± 1.96 ms for the fifth step in maximum sprints [23]. In a study on the maximum velocity phase, the mean differences were −2.5 ± 4.8 ms (LOA: −11.8 to 6.8 ms) at an algorithm detection rate of 95.7% [19], while another study reported mean differences within ± 5 ms with LOAs at approximately 15–30 ms for elite and amateur sprinters [24]. Machine or deep learning approaches for GC detection detected 97% of GCs during different gait tasks without quality assessment using a Beta Process Auto Regressive Hidden Markov Model [25]. A bidirectional long short-term memory network achieved 5 ± 42 ms (mean differences and LOA) in comparison with GC times from force-sensing insoles at running speeds ranging from 2 to 6 m/s; IC and TC detection exhibited higher variability at increased speeds [26]. Machine learning algorithms have not yet been comprehensively validated for sprint-specific analyses, although they demonstrate comparable accuracy relative to heuristic-based approaches.

Deep learning architectures, such as convolutional neural networks (CNNs) and long short-term memory networks (LSTMs), are particularly well-suited for high-dimensional or time-series data and have demonstrated promising performance in time-series classification (TSC) tasks [27]. CNNs can be defined as both convolution-based and as a class of deep learning-based algorithms for TSC when the deep learning architecture contains convolutions [28]. These CNNs have already been used in TSC for EMG pattern recognition [29], snowboard halfpipe jump detection [30], or lower limb joint kinematics estimation in gait [31,32]. One-dimensional CNNs (1D-CNN) are the most prominent and suitable for extracting temporal information [33]. Different CNN architectures exist, e.g., U-Net, fully convolutional network, ResNet, or InceptionTime [30,34]. Although there is rapid development in this area, inception-based models like InceptionTime architectures are widely used and have been shown to have superior performance for many TSC tasks [28,33,34]. However, CNNs with this architecture have not yet been applied to IMU time series in sprinting.

The purpose of this study is to evaluate the potential of a deep learning 1D-CNN for detecting GC events (IC and TC) and GC times, with a specific focus on the acceleration phase of athletic sprints, which has not yet been examined using deep learning methods.

## 2. Materials and Methods

### 2.1. Subjects

Twelve sprint-trained athletes (19.7 ± 3.7 yrs; 1.78 ± 0.11 m; 68.6 ± 9.5 kg; 6 female, 6 male; 100 m personal bests of 11.30 ± 0.42 s (male) and 12.19 ± 0.48 s (female)) completed three 60 m sprints off the starting block interspersed with 20 min rest intervals. Athletes’ velocity development was measured with a 1080 Sprint 2 (1080 Motion Inc., Austin, TX, USA) with a 1.5 kg minimum resistance attached at the hip of the athlete; however, velocity was not used in this study. The first 15 m of the 60 m sprints were analyzed, which equals the phase of highest acceleration [35] while capturing the initial acceleration phase and parts of the acceleration transition phase [10,11,12,14,15,16,17]. The tests were integrated into the athletes’ regular training schedule, with their coach supervising pre-run preparations. Informed consent was obtained from all athletes prior to their participation. The study was conducted in accordance with the Declaration of Helsinki, and the procedures were approved by the Otto von Guericke University Ethics Committee (Reference number: 27/25).

### 2.2. Data Collection and Preparation

First, 6DOF gyroscope and accelerometer data were collected from two IMUs (Vicon Motion Systems Ltd., Oxford, UK) at 1125 Hz. IMUs were attached laterally to the left and right shanks using latex straps. Resultant acceleration (a_RES_) and angular velocity (ω_RES_) data were computed from the raw tri-axial IMU (x, y, z) data and subsequently resampled to 250 Hz. Three synchronized high-speed cameras (Baumer Holding AG, Frauenfeld, Switzerland) positioned at 0, 5, and 10 m captured video footage at 250 fps from a 90° sagittal perspective.

The IMU dataset consisted of two sensors per athlete, yielding a total of 70 athlete–run–leg combinations. Data from run 3 of athlete 1 were excluded due to a sensor data download failure. IMU data were trimmed to the running phase, from block release to after the final contact, based on holistic known IMU GC patterns. In the 250 fps high-speed videos, the GCs, defined as the first and last frames showing foot contact with the ground, were identified for each run. The video GCs were converted into a binary time series ∈ {0, 1}, where 1 indicated GC, and 0 indicated the flight phase. For IMU–video synchronization, the maximum vertical heel drop was defined as the moment immediately following touchdown when the heel exhibited its greatest downward displacement between consecutive video frames. This event was assumed to correspond to a peak in a_RES_ recorded by the shank-mounted IMU. All identified GC events were subsequently cross-correlated between IMU and video time points to ensure precise temporal alignment across the entire sprint trial, even in the presence of minor measurement noise or labeling inaccuracies in individual GC events. The dataset of synchronized binary video GCs and IMU data per run was used for the training of the 1D-CNN.

### 2.3. One-Dimensional CNN

In general, 1D-CNNs have been shown to be successful in IMU event detection [18,29,30]. In a comparison of different 1D-CNN architectures, the InceptionTime architecture consistently showed superior performance for TSC [28,36].

**Figure 1:**
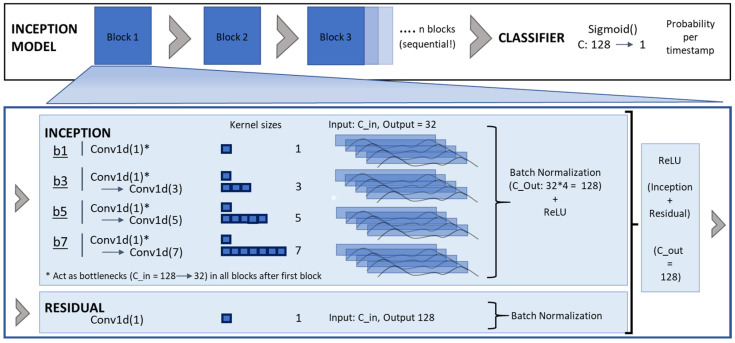
The 1D-CNN inception model flow chart. Sequential inception blocks. Each block has an inception and a residual part. C refers to feature input channel dimensions. The first block, C_in is always two features; for all other blocks, C_in is 128. For the inside blocks, the branch (b1, b2, b3, and b4) output is always 32, which gets concatenated to a total block output of C_Out = 128.

The InceptionTime model is a deep neural network for TSC, built from three stacked inception blocks, each containing three inception modules *I*(*x*) and, for stable calculation, a residual block, *R*(*x*) [27]. The concept of these inception blocks was used for the proposed model. It was built with one inception module *I*(*x*) and its corresponding residual module *R*(*x*) per block (Figure 1). The model consisted of several subsequent blocks, resulting in the following formulas for each block output:

(1) $$R(x)=BN(W_{r}*x),$$

(2) $$I(x)=BN({[b}_{1}(x)\;\|\;b_{3}(x)\;\|\;b_{5}(x)\;\|\;b_{7}(x)]),$$

(3) $$y=ReLU\;\left(I\left(x\right)+R\left(x\right)\right),$$

In the InceptionTime, each inception module *I*(*x*) consists of three parallel convolutional branches that capture temporal features at different receptive field sizes (kernel) [27]. To expand this feature in the proposed model, four kernels with sizes k ∈ {1, 3, 5, 7} were applied to improve capture of both short- and long-term characteristics in the IMU data (4).

(4) $$b_{1}(x)={W\;}_{k,1}\;*\;\;x,\;\;\;\;\;\;\;\;\;k\;\in \;\{1\},$$

(5) $$b_{k}(x)=W_{k,2}\;*\;(W_{k,1}\;*\;x),\;\;\;\;\;\;\;k\;\in \;\{3,\;5,\;7\},$$

In the first block, the model receives two input channels, a_RES_ and ω_RES_ (C = 2). Each branch expands this to 32 channels [27] to obtain a more detailed resolution for the explicit convolutional kernels to detect underlying patterns (Figure 1). The outputs of the four parallel branches are concatenated, yielding 128 channels per time step. To maintain dimensional consistency for the residual connection, the block input is projected via a 1 × 1 convolution (4) $W_{r}\in R^{128\times 2\times 1}$ [27] followed by batch normalization (Figure 1). This projected residual *R*(*x*) is then summed up with the output of the inception module *I*(*x*). The final block output is obtained via a ReLU activation (3). In subsequent blocks, the $W_{k,1}$ convolutions (4, 5) within each branch act as bottlenecks, which comprise the input from the dimension of 128 to 32 for reduced computational effort and more dense pattern extraction, before the branch-specific temporal convolutions (Figure 1) [27].

After all subsequent inception blocks, a classifier is applied to generate the output probabilities at each individual timestep (Figure 1). All convolutional operations are one-dimensional and applied along the temporal axis so that each time step is transformed independently across channels while preserving the sequence length. The lightweight classifier consists of a pointwise 1 × 1 convolution $W_{C}\in R^{1\times 128\times 1}$ followed by a sigmoid activation (6). It maps the 128 per-timestep channel feature representation of the last inception block to a single probability per timestep.

(6) $$\hat{y}=sigmoid(\;W_{C}\;*\;y)$$

### 2.4. Dataset

The collected synchronized binary video GC–IMU dataset per run was used for model training. Video GCs served as ground-truth (GT) labels for GC. Input features were the synchronized a_RES_ and ω_RES_ IMU channels (C = 2). CNNs generally require fixed-length inputs, but the complete runs have different time-length data [36]. While different approaches like padding, truncation, or normalizing exist to solve the time-length issue, they all compromise the original data [36]. Gorges et al. (2024) showed that slicing the data into windows is a successful approach to avoid fixed-length problems. They also stated that the window size should be hyperparameter-tuned relative to the classification task of the model [30]. Therefore, features and labels were split into fixed-length windows that overlapped with a certain stride, and values for these parameters were defined during hyperparameter training. The i-th window is

(7) $$X^{\left(i\right)}={\left[x_{i},x_{i+1},\ldots ,x_{i+T-1}\right]}^{⊤}\in R^{2\times T},$$

(8) $$y^{\left(i\right)}=\left[y_{i},y_{i+1},\ldots ,y_{i+T-1}\right]\in \{0,1{\}}^{T},$$

Incomplete windows at the end of runs (<window size T) were discarded to ensure fixed-length input segments for model training. The features were min–max scaled to the range [−1.5, 1.5] for each run to ensure comparability across runs, steps, leg sides, and athletes due to large variations in touchdown peak magnitudes. The final dataset is

(9) $$X\in R^{N\times 2\times T},\;\;Y\in \{0,1{\}}^{N\times T},$$

with *N* representing the total number of windows across all athletes, runs, and leg sides. For evaluation, the model-predicted probabilities were reassembled into the original continuous time series by averaging values across overlapping window strides.

### 2.5. Dataset Split

In biomechanics, model performance needs to be assessed not only in controlled laboratory settings but also for field scenarios, making a split into training, validation, and test datasets essential [37]. The dataset was partitioned on an athlete- and gender-specific basis. Data from athletes 2–11 were used for training and validation. Due to the limited number of athletes in the test protocol, only one male and one female athlete (1 and 12) were reserved exclusively as unseen test subjects. Within the training–validation group, runs 1 and 2 were assigned to training (TRAIN) and run 3 to validation (VAL). The test dataset (TEST) contained the two available runs of athlete 1 and all three runs of athlete 12. This design enabled the evaluation of the model’s generalization to both familiar (VAL) and unseen (TEST) athlete-specific movement patterns for GC detection from IMU signals.

### 2.6. Model Training and Hyperparameter Tuning

In total, the TRAIN consisted of 40 IMU data streams (10 athletes × 2 runs × 2 legs) of synchronized a_RES_ and ω_RES_ signals, each paired with their respective ground-truth labels. Hyperparameter tuning was performed using *Weights & Biases* (W&B, San Francisco, CA, USA) to optimize model performance. The tuned parameters included window size ∈ [25, 50, 75, 100, 150, 200], stride ∈ [5, 10, 15, 20, 25, 30], number of subsequent inception blocks ∈ [6, 9, 12, 15, 18, 21], and learning rate ∈ [0.01, 0.001, 0.0001]. Combinations with stride ≥ window size were skipped. The tuning objective was to minimize the mean Hausdorff distance for the VAL. Training was performed using the Adam optimizer for a maximum of 30 epochs and a batch size of 50. Early stopping was applied based on the validation binary cross-entropy loss, terminating training if the loss did not improve for five consecutive epochs to avoid overfitting.

### 2.7. Evaluation and Metrics

Model performance was evaluated on a VAL (10 athletes × 2 legs = 20 datastreams) and the independent TEST set (10 datastreams) predictions. Model probability outputs were binarized (PRED) using a threshold of 0.5, with probabilities below 0.5 classified as no GC = 0 and those equal to or above 0.5 as GC = 1. GC detection was assessed from two aspects. First, transitions between flight and contact were evaluated. These were calculated as turning points from 0→1 and 1→0 in the binary GT or PRED. The mean and median Hausdorff distances (in frames) were computed between predicted and true transitions (=IC or TC events). Second, GC times were defined as continuous periods where the signal equaled 1 for GT and PRED, respectively (duration from IC until TC). Precision, recall, and the Rand Index were computed for each data stream, with precision and recall based on GCs as events and the Rand Index representing agreement in GC time (in frames).

Metrics were computed before and after a postprocessing step. As noted by Gorges et al. [30], 1D-CNN outputs for TSC can occasionally produce very short false prediction spikes. These may mislead Hausdorff metrics and lead to incorrect interpretations. To address this, predicted GCs shorter than 12 frames (48 ms), which are approximately half of the shortest elite 100 m sprinting GC times (>90 ms) [20] and therefore cannot represent an actual athlete foot touchdown, were converted to non-predictions (binary = 0). Additionally, incomplete window predictions at the start or end of each data stream were reset to 0 to ensure clean predictions across the run.

### 2.8. Statistics

Following model evaluation, the deep learning approach for GC detection was statistically benchmarked against the video-based ground truth using only the postprocessed model predictions. Stepwise mean GC times for GT and PRED were reported using descriptive statistics (mean ± SD). Normality (Shapiro–Wilk) and homogeneity (Levene’s) tests indicated non-normal distributions for VAL GT, VAL PRED, and TEST GT, leading to the use of non-parametric tests. Differences were examined using the Wilcoxon signed-rank test (*p* < 0.05), with effect sizes (ES) interpreted as <0.2: trivial, 0.2–0.49: small, 0.5–0.79: medium, and ≥0.8: large [38]. Bland–Altman plots were constructed to examine systematic and proportional bias for GC time, with heteroscedasticity defined as *r*^2^ > 0.1; mean differences + LOA and mean absolute deviations ± SD were reported [39]. IC and TC event detection accuracy in GT and PRED was examined in boxplots. Concurrent GC time validity was assessed using Spearman’s correlation (ρ < 0.1: trivial, 0.1–0.3: small, 0.3–0.5: moderate, 0.5–0.7: large, 0.7–0.9: very large, >0.9: almost perfect) [40]. Finally, linear regression plots supported by mean absolute percentage error (MAPE) and root mean square error (RMSE) parameters illustrate the linear relationship between the model GC times PRED and the GT measurements. All statistical analyses were performed in Python, version 3.13.7 (including SciPy Stats library).

## 3. Results

The results of the 1D-CNN model, its performance metrics, and the statistical evaluation of the model as a tool for defining GCs in sprint acceleration are presented here.

### 3.1. Hyperparameter Tuning

Hyperparameter optimization revealed that the model achieved optimal predictive performance when configured with 18 inception blocks, using input windows of 100 data points and a stride of 15 (Table 1).

### 3.2. Model Metrics

The performance of the model with respect to the TEST and VAL data was assessed using different metrics (Table 2).

A recall of 1 signifies a 100% correct GC event detection for VAL and TEST (Table 2). The TEST precision, with a value slightly below 1, indicated a very small number of false positives for predicted GC events. These also negatively influenced the Hausdorff values. Postprocessing successfully eliminated the false positive predictions, and mean and median Hausdorff distances were ≤1.5 frames (≤6 ms) for both TEST and VAL. The Rand Index exceeded 0.977, demonstrating near-perfect alignment between predicted and true GC times. As expected, the Rand Index was unaffected by postprocessing, since this procedure does not alter correctly predicted contact segments. All subsequent analyses were conducted on the postprocessed data.

### 3.3. Performance Values

The GC times exhibited a clear trend of progressively shorter durations with increasing step number (Table 3). The largest deviations in mean GC times were observed for the first, fourth, eighth, and ninth steps, whereas the remaining steps showed comparatively small variations, indicating overall consistency across the sprint sequence. GT values were generally lower in the TEST, which also displayed the shortest mean GC time of 98 ± 4 ms at step 9 (Table 3).

### 3.4. GC Detection Method Evaluation

**Figure 2:**
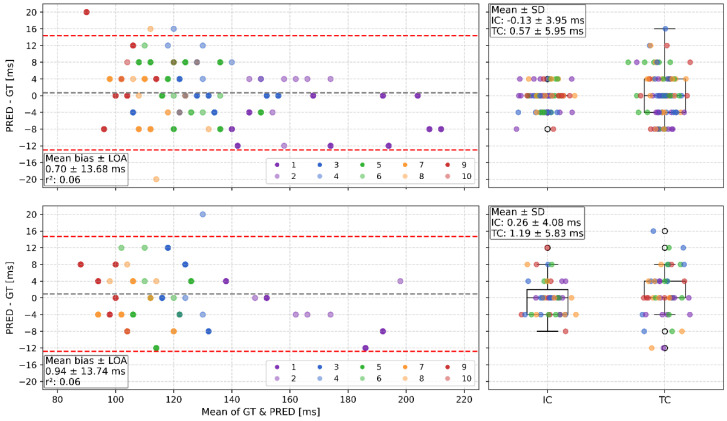
VAL Bland–Altmann and boxplots (top) and TEST Bland–Altman and boxplots (bottom). Individual points are color-coded according to the step. Bland–Altman plots show systematic bias between GT and PRED. Boxplots present deviations from PRED vs. GT for IC and TC events. Both systems were synchronized and sampled at 250 Hz, yielding a temporal resolution of 4 ms per data point. This discrete resolution explains the grid-like points pattern (± 0, 4, 8, 12, 16, and 20 ms).

**Figure 3:**
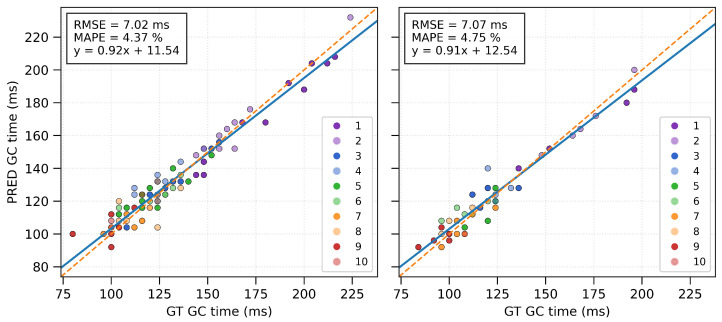
Left (VAL) and right (TEST) plots illustrate the linear relationship between the model predictions and the ground-truth GC measurements obtained from video recordings. Individual points are color-coded according to the step index, while the dashed line represents the ideal identity line. Both systems were synchronized and sampled at 250 Hz, yielding a temporal resolution of 4 ms per frame. This discrete resolution explains the grid-like point pattern.

The Bland–Altman figures for GC times revealed similar mean differences below 1 ms and LOA around +15 and −13 ms for both VAL and TEST conditions (Figure 2, left), which were not statistically significant (Table 4). The boxplots showed a lower mean and standard deviation for differences between PRED and GT for IC than in TC events in both VAL and TEST (Figure 2, right). Between GT and PRED in both settings, the correlations were almost perfect (>0.90), and the RMSE was around 7 ms across the varying GC times (Figure 3). The mean absolute error of GC time was around 5–6 ms (<5% MAPE) in both VAL and TEST.

## 4. Discussion

A deep learning 1D-CNN based on inception modules was trained to assess the detection of GC events and GC times during the acceleration phase of maximum sprinting.

The model was hyperparameter-tuned for window size, stride, inception blocks, and learning rate. The model performed best with 18 blocks, the second highest of all possible blocks ∈ [6, 9, 12, 15, 18, 21] for the hyperparameter tuning, combined with the greatest learning rate of 0.01. With 18 blocks, the model apparently benefited from deep learning for better performance. The window parameters were tuned to an optimum at a size of 100 data points and a stride of 15. Compared to the average contact time of athletes in this study (VAL: 132 ± 30 ms, TEST: 123 ± 31 ms), the window represents more than triple GC time at the measured 250 Hz sampling rate (100 data points equal 400 ms). Visual inspection of the complete run data (Appendix A.2 and Appendix A.3) indicates that the 100 data points window might correspond more closely to the full step cycle, encompassing both GC and flight phases. Gorges et al. (2024) pre-defined their window size according to the length of the classification task and achieved accurate results [30] while recommending hypertuning of the parameter. This study confirms that selecting a window size that captures the full cycle of the task may be beneficial for the model. Looking at the metrics mean and median Hausdorff, precision, and Rand Index, the suggested model performs very well for GC event classification in time-series, although the TEST needed postprocessing to optimize the mean Hausdorff distance. The VAL was relatively stable in the context of hyperparameter optimization, with the objective to minimize the mean Hausdorff distance. Nevertheless, it could be advisable to apply the preprocessing step prior to the computation of the optimized metric. This ensures that model selection is based on the most representative evaluation of predictive performance, rather than being influenced by easily removable false predictions.

The model achieved mean differences of 0.70 with LOA 14.39 to −12.98 ms in the VAL and 0.94 with LOA 14.68–12.81 ms in the TEST. Their mean absolute deviations were 5.36 ± 4.55 ms (VAL) and 5.53 ± 4.35 ms (TEST), reflecting a <5% MAPE for GC time prediction in both VAL and TEST, thus providing reliable measurement accuracy. The IC and TC boxplots (Figure 2) reveal that the remaining deviations are based more on inaccuracies in the TC detection, which is consistent with the previous literature [5,25]. In comparison with heuristic algorithms for GC detection in sprinting, the model performs similarly to or better than current research. Across all running phases in elite sprinters’ 100 m runs, mean absolute time deviations of 5.46 ± 4.55 ms were observed [20]. In a 50 m maximum accelerated sprint, two IMU systems with sampling frequencies of 240 Hz and 1000 Hz had a significant mean bias of 4 ms (trivial correlation) and 3 ms (moderate correlation) for the step-specific mean GC times compared against force plates [22]. Research for the maximum speed phase showed mean differences of −2.5 ± 4.8 ms (LOA: −11.8 to 6.8 ms) [19] or within a range of ±5 ms and LOA approximately at 15–30 ms for elite and amateur sprinters [24]. A focused analysis of the acceleration phase reveals that the proposed model achieves performance similar to or superior to previously reported approaches. Purcell et al. (2006) reported −8 ± 17.64 ms for the first step, −2 ± 9.8 ms for the third, and 0 ± 1.96 ms for the fifth step in maximum sprints [23], and Miranda-Oliveira et al. (2023) reported mean deviations of −71 ± 70.3 ms (LOA) for steps within the first 10 m [21]. A machine learning algorithm (bidirectional LSTM) also performed worse than the proposed algorithm, with a 5 ± 42 ms mean difference and LOA [26].

The proposed model achieved a GC event detection rate of 100% in both the VAL and TEST datasets after postprocessing. To the best of the authors’ knowledge, no previous study has reported a comparable detection rate for GC events in sprinting. The reference detection rates range between 93 and 98% over all phases [19,20,21,23]. Furthermore, detection rates within the initial acceleration phase, as examined in this study, are typically even lower, e.g., missing 6.47% for the first five steps or 7% in the first 10 m [20,21]. Even machine learning approaches specifically developed for GC detection, rather than for GC time accuracy optimization, have reported failure rates of around 3% [25].

Heuristic approaches, which rely on pattern interpretation within IMU signals, cannot ensure that the detected events truly correspond to those in the reference system. This limitation arises from the athlete-specific movement techniques [19], the segment of IMU attachment, and especially varying or changing speed levels, where minima, maxima, thresholds, and time windows may fluctuate [5]. Deep learning models based on a synchronized video reference used for model training could provide a better basis for correct GC detection, especially in high-speed sprinting. In future studies, this can further be enhanced by athlete-individual model refinement. High-speed video, alongside force plates with a low one-step threshold, represents the current gold standard as a reference tool for GC event detection [22]. Caution must be taken when using photoelectronic methods in elite sports, as they introduce a physically induced error by measuring the foot crossing a photoelectronic barrier, which is always a certain distance above ground, rather than measuring the actual GC. Ultimately, the accuracy and the preferred reference method are likely to be dependent on the running surface and might differ between track and field, team sports with natural or artificial grass, indoor sports halls, or even iced surfaces, as in bobsleigh or skeleton.

Accurate GC time measurement is more important in maximum effort sprinting than in regular running or gait, as GC times are shorter. Minimum mean GC time was 94 ± 6 ms in the TEST at step 9 and can come down to 90 ms in elite sprinters [20]. For higher accuracy, the sampling frequency appears to be crucial. Frame-perfect detections of both IC and TC events mean the true event is within ±2 frames if it is at the edges of the time between the two event-defining frames (up to one frame too early for IC and one frame too late for TC). For a 94 ms GC, this equals an error percentage up to 2/sampling frequency step (in ms)/94 ms. The true GC time error percentage is therefore less but up to 21.3% at 100 Hz, 8.5% at 250 Hz, 4.3% at 500 Hz, and 2.1% at 1000 Hz at frame-perfect detection. To ensure values within a 10% error range in short GC times < 100 ms, as observed in maximum sprinting and at the elite level, it is recommended that at least 200 Hz (5 ms frame-to-frame) be used for measurements. This study’s sampling rate for both video and IMU was 250 Hz, incorporating 4–8 ms (<8.5%) inaccuracies at the frame-exact level.

The synchronization approach employed in this study was based on the interpretation of manually labeled foot events and corresponding sensor movement during running. Although the applied multi-step cross-correlation method eliminates systematic synchronization errors, it cannot guarantee perfect alignment at a sampling rate of 250 Hz due to possible unsystematic labeling errors at the frame level. These errors additionally likely affect model performance, as they unsystematically influence reference GT data points for model learning. Future studies should aim to ensure a more precise synchronization task or implement a dedicated technical synchronization mechanism, although the latter is a challenge for portable systems like IMUs, as most wireless systems contain a certain degree of signal delay.

The deep learning 1D-CNN model applied in this study was based on using inception blocks [27]. The performance was better than previously used ML models; however, the computational cost of the proposed (or any deep learning) model is referenced as comparably high [28]. Inference runtimes of 0.221 ± 0.007 s for processing all inference windows (corresponding to 0.117 ms per window) and a throughput of 8516 windows·s^−1^ indicate that the proposed model is suitable for field deployment; however, these performance characteristics were achieved using an NVIDIA RTX 2000 Ada Generation Laptop GPU, with a peak GPU memory allocation of 468.8 MB and a peak reserved memory of 610.0 MB (Appendix A.1). As the literature in TSC is evolving very fast [28], future models could be used with less computational effort while delivering the same accuracy results. This would allow an easier transfer to real-world applications, which should be the ultimate goal of any GC detection algorithm and IMU usage in applied sprinting, although most are currently only used indoors or for treadmills [6].

The model was trained on data from different athletes and different steps. Athletes’ sprinting biomechanics may have been slightly affected by the 1.5 kg resistance applied by the attached 1080 Sprint device for velocity measurements. While the 1.5 kg towing resistance might have slightly altered sprint mechanics, we expect its effect on the relative timing of GC events to be minimal. Owing to the measurement settings with a small intra-athlete sample size, the data is not large enough to fully conduct an athlete- or step-specific statistical analysis. The small intra-athlete sample size also limited the TEST dataset to only two athletes, possibly influencing the results if the model captured athlete-specific performance characteristics. Future research could build on this and conduct research on higher sample sizes to gain further insights on athlete- or step-specific patterns and model prediction accuracies while confirming the findings under fully free sprinting conditions.

## 5. Conclusions

The proposed model, after postprocessing, achieved a 100% GC detection rate in the acceleration phase of a sprint, outperforming both heuristic and previously published machine learning approaches. The observed mean absolute deviations and limits of agreement match the values reported in current state-of-the-art GC time detection studies over all sprinting phases and surpass the values reported specifically for the more challenging condition of the acceleration phase. The model additionally maintained consistent performance between the VAL and TEST datasets, suggesting methodological robustness and transferability to field application. To enhance model generalization in field applications, future studies should overcome the current limitations by employing broader datasets, multiple sensor configurations, and fully free sprinting conditions. Additionally, with ongoing advances in time-series classification models, researchers should remain attentive to emerging architectures that offer comparable accuracy with greater computational efficiency.

In summary, the findings highlight the strong potential of deep learning-based time-series models for IMU-driven GC detection in the acceleration phase of sprinting. Such models represent a promising step toward reliable, field-applicable biomechanical monitoring tools for sprint performance analysis.

## Figures and Tables

**Table 1:** The 1D-CNN hyperparameters.

Parameter	Optimized Value
window size	100
stride	15
number of blocks	18
learning rate	0.01

Optimized hyperparameters of the model after W&B tuning.

**Table 2:** Model metrics.

Data	Mean Hausdorff		Median Hausdorff		Precision	Recall	Rand Index
VAL	1.3 ± 1.38	(5.2 ± 5.52)	1 ± 0.71	(4 ± 2.84)	1 ± 0	1 ± 0	0.980 ± 0.015
VAL pp	1.3 ± 1.38	(5.2 ± 5.52)	1 ± 0.71	(4 ± 2.84)	1 ± 0	1 ± 0	0.980 ± 0.015
TEST	9.4 ± 16.69	(37.6 ± 66.76)	2 ± 12.44	(8 ± 49.76)	0.96 ± 0.07	1 ± 0	0.977 ± 0.018
TEST pp	1.5 ± 0.67	(6 ± 2.68)	1 ± 0.60	(4 ± 2.4)	1 ± 0	1 ± 0	0.977 ± 0.018

Model metrics averaged per run and afterwards averaged across dataset condition for the two datasets VAL and TEST without and with postprocessing (pp). Metrics are presented as mean ± standard deviation (mean Hausdorff, precision, recall, and Rand Index) and median + median absolute deviation (median Hausdorff). Hausdorff distances are represented in frames (ms in brackets).

**Table 3:** GC times.

Step	1	2	3	4	5	6	7	8	9	10	Total Steps
Data											
Overall GT	184 ± 30	175 ± 24	134 ± 16	132 ± 13	124 ± 13	122 ± 11	115 ± 12	114 ± 10	104 ± 12	104 ± 5	134 ± 31
VAL GT	181 ± 26	170 ± 24	132 ± 16	131 ± 14	125 ± 14	122 ± 10	114 ± 11	114 ± 11	103 ± 10	102 ± 2	132 ± 30
VAL PRED	175 ± 27	171 ± 26	132 ± 16	136 ± 11	126 ± 11	123 ± 6	113 ± 11	114 ± 8	107 ± 9	106 ± 2	132 ± 28
TEST GT	166 ± 23	170 ± 16	122 ± 8	122 ± 6	115 ± 6	110 ± 10	107 ± 9	106 ± 7	94 ± 6	108 ± 0	123 ± 31
TEST PRED	162 ± 18	169 ± 17	123 ± 5	129 ± 6	115 ± 9	115 ± 5	103 ± 8	110 ± 5	98 ± 4	102 ± 2	124 ± 26

Step-specific GC times (ms) are reported as mean + standard deviation comparing ground-truth (GT) video data to model-predicted (PRED) values for the overall dataset, VAL, and TEST after postprocessing. The number of steps per condition was as follows: steps 1 to 8: overall = 35, val = 10, test = 5; step 9: overall = 29, val = 9, test = 5; step 10: overall = 9, val = 2, test = 2; total steps overall = 318, val = 91, test = 47.

**Table 4:** GC statistics.

	Mean Differences	Mean Absolute Differences	Spearman r	Spearman *p*	Difference Testing *p*	Difference Testing ES
VAL	0.70 ± 6.98	5.36 ± 4.55	0.941	<0.001	0.415	0.423
TEST	0.94 ± 7.01	5.53 ± 4.35	0.920	<0.001	0.395	0.414

GC times mean differences (ms), mean absolute differences (ms), correlations, and difference testing results for the VAL and TEST datasets between GT and PRED. Samples numbers: VAL = 91, TEST = 47 for GT and PRED, respectively.

## Data Availability

The athlete data presented in this study are available upon reasonable request from the corresponding author due to privacy and ethical reasons. The 1D-CNN model code is provided at https://github.com/DoloCode/DeepLearningCNN-BasedGroundContactDetectionDuringSprintAcceleration, last accessed on 29 November 2025.

## Abbreviations

The following abbreviations are used in this manuscript:

GC	Ground contact
IC	Initial contact
TC	Terminal contact
IMU	Inertial measurement unit
1D-CNN	One-dimensional convolutional neural network
CNN	Convolutional neural network
LSTM	Long short-term memory network
TSC	Time-series classification
a_RES_	Resultant acceleration
ω_RES_	Resultant angular velocity
GT	Ground Truth
PRED	Prediction
TRAIN	Training dataset
VAL	Validation dataset
TEST	Test dataset
pp	postprocessed
ES	Effect size
LOA	Limits of agreement

## Appendix A

### Appendix A.1. Model Inference Time, Computational Load Information, and Hardware Specifications

**Category**	**Metric**	**Value**
Hardware	GPU	NVIDIA RTX 2000 Ada Generation Laptop GPU
	CUDA version	11.8
	PyTorch version	2.7.1 + cu118
Model size	Trainable parameters	0.85 M
Computational load	FLOPs per inference window	0.085 GFLOPs
	Effective throughput	0.726 TFLOPs
Inference runtime	Total inference time (mean ± SD)	0.221 ± 0.007 s
	Inference time per window	0.117 ms
	Throughput	8516 windows·s^−1^
Memory usage	Peak GPU memory allocated	468.8 MB
	Peak GPU memory reserved	610.0 MB

Inference time was measured as wall-clock time using CUDA synchronization and averaged over repeated runs following warm-up iterations. GPU memory usage was obtained using PyTorch CUDA memory statistics. Effective throughput was computed from measured inference time and model FLOPs.
